# Nonunion of Isolated Medial Cuneiform Fracture Fixed With a Compression Screw and Compression Staple: A Case Report

**DOI:** 10.7759/cureus.58074

**Published:** 2024-04-11

**Authors:** Munekazu Kanemitsu, Tomoyuki Nakasa, Katsunori Shiraishi, Yasunari Ikuta, Nobuo Adachi

**Affiliations:** 1 Department of Orthopaedic Surgery, Akiota Hospital, Hiroshima, JPN; 2 Department of Artificial Joints and Biomaterials, Graduate School of Biomedical and Health Sciences, Hiroshima University, Hiroshima, JPN; 3 Department of Orthopaedic Surgery, Matsuyama Shimin Hospital, Matsuyama, JPN; 4 Department of Orthopaedic Surgery, Graduate School of Biomedical and Health Sciences, Hiroshima University, Hiroshima, JPN

**Keywords:** fracture, compression screw, medial cuneiform, #nonunion, compression staple

## Abstract

Isolated cuneiform fractures are rare and account for only 1.7% of all midfoot fractures. Medial cuneiform fractures can be treated conservatively or surgically, with good clinical outcomes. However, nonunion is a rare complication of medial cuneiform fractures, and only a few cases have been reported in the literature. We report a case of a medial cuneiform fracture requiring surgical treatment that had a good clinical outcome. A 15-year-old boy presented to an orthopedic clinic with a complaint of pain in his right foot. The patient had landed on the foot during a handball game and was treated conservatively for several months. However, his symptoms persisted, and he was referred to our clinic for further evaluation, where he was diagnosed with medial cuneiform nonunion of the right foot. Open reduction and internal fixation surgery using a compression screw and staple and autologous bone grafting were performed. Postoperatively, bone union was observed, and the patient returned to full competition with no complaints of pain during exercise. The Self-Administered Foot Evaluation Questionnaire (SAFE-Q) score at 21 months after surgery was 100.0 for the following subscales: Pain & Pain-Related; Physical Functioning & Daily Living; Social Functioning; Shoe-Related; General Health & Well-Being; and Sport (handball). We encountered a case of an isolated medial cuneiform fracture that required surgical treatment. During the surgical treatment, fixation with a combination of compression staples and screws may be considered simple and useful for achieving strong fixation because the medial cuneiform fracture has a small bone fragment.

## Introduction

The medial cuneiform bone, along with the other cuneiforms, metatarsal, and cuboidal bones, forms the Lisfranc joint and is involved in the formation of the transverse arch. Isolated cuneiform fractures are rare, accounting for approximately 1.7% of all midfoot fractures [1]. Cuneiform fractures are often associated with other injuries, such as fractures of adjacent cuneiforms, or are part of a larger injury complex, such as Lisfranc fracture-dislocations [2, 3]. Of the reported cases, several occurred due to high-energy trauma or a direct blow to the dorsal midfoot [1, 3-12]. Medial cuneiform fractures can be treated conservatively or surgically, with good clinical outcomes [3]. However, nonunion is a rare complication of medial cuneiform fractures, and only a few cases have been reported in the literature [4, 10]. Herein, we report a case of medial cuneiform fracture that required surgical treatment in which a good clinical outcome was achieved using a compression screw and staple.

## Case presentation

A 15-year-old boy presented to an orthopedic clinic with complaints of pain in his right foot. The patient had landed on the foot during a handball game. No abnormal findings were noted on radiography, and the patient’s foot was immobilized in a cast for several weeks. However, his symptoms did not improve, and his right-foot pain persisted during sports activities. Three months after the injury, he consulted another orthopedic clinic, and nonunion of the medial hallux was noted. The patient was referred to our clinic for further evaluation.

**Figure 1:**
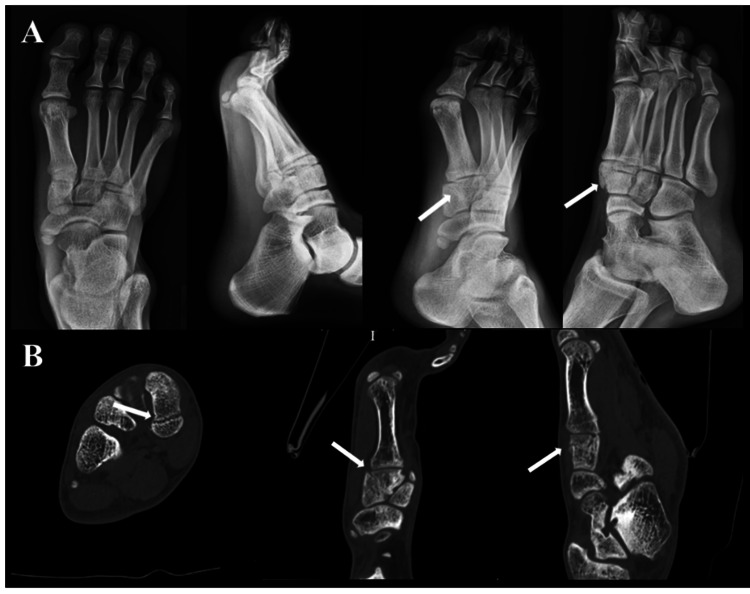
Preoperative images. A: Radiograph; B: CT (White arrows indicate the fracture line)

Tenderness was also observed in the medial cuneiform. Radiography and CT showed approximately 2 mm displaced oblique fracture of the medial cuneiform bone and sclerotic changes in the fracture line (Figure 1). Self-Administered Foot Evaluation Questionnaire (SAFE-Q) [13-15] scores were 91.7 for Pain & Pain-Related, 97.7 for Physical Functioning & Daily Living, 83.3 for Social Functioning, 100.0 for Shoe-Related, 100.0 for General Health & Well-Being, and 22.2 for Sports (Handball).

**Figure 2:**
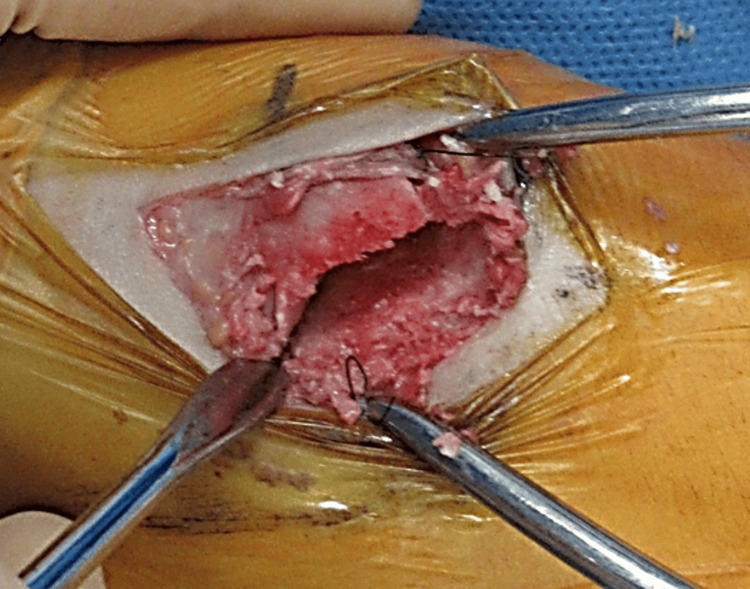
Operative findings. Nonunion of medial cuneiform fracture was observed.

**Figure 3:**
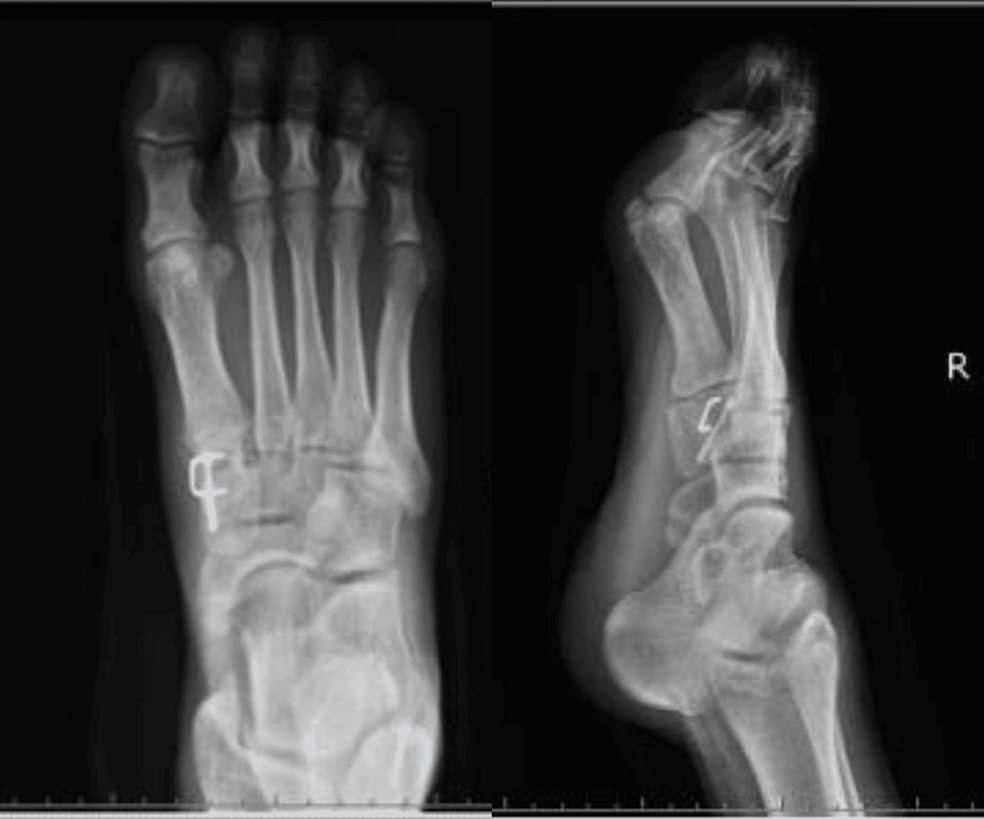
Postoperative images. The fracture was fixed with a headless compression screw and staple.

The patient was diagnosed with medial cuneiform nonunion of the right foot. He was placed in the supine position under general anesthesia, and surgery was performed. An approximately 5 cm skin incision was made directly over the fracture site, and the fractured bone was exposed. The fracture was adjacent to fibrous scar tissues that could be debrided (Figure 2). The fracture was redressed using a scalpel and sharp spoon, and bleeding was observed. An approximately 3 cm long skin incision was then made at the medial malleolus. The periosteum was dissected, the cortical bone was opened in an area of approximately 7 × 7 mm, and the cancellous bone was harvested. The harvested cancellous bone was grafted into the nonunion site and fixed with a ø 2.5 mm headless compression screw and 11 mm × 10 mm compression staples (Figure 3).

**Figure 4:**
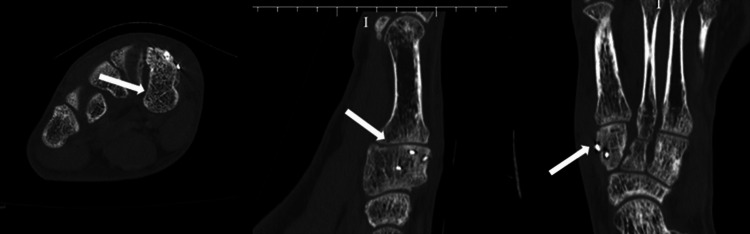
Postoperative CT at 3 months after the operation. Bone union was observed.

The patient was immobilized using a cast for 3 weeks postoperatively. Four weeks after surgery, partial weight bearing was allowed with an insole, and full weight bearing was allowed 7 weeks after surgery. Three months postoperatively, bone union was observed on CT (Figure 4), and the patient was allowed to return to sports. SAFE-Q scores at 21 months after surgery were 100.0 for all the subscales: Pain & Pain-Related; Physical Functioning & Daily Living; Social Functioning; Shoe-Related; General Health & Well-Being; and Sport (handball). The patient returned fully to participating in competitions with no complaints of pain during exercise.

## Discussion

Medial cuneiform fractures are usually part of a more complex injury involving the foot and ankle; therefore, isolated medial cuneiform fractures are rare [2], and since most cases are treated conservatively, there is no established surgical technique. The diagnosis of medial cuneiform fractures, especially nondisplaced fractures, is difficult because of the complex nature of the anatomical structures of the foot bones; these fractures may be overlooked leading to a delay in treatment [9]. Since radiography may not be adequate when a medial cuneiform fracture is suspected, CT and MRI should be used for diagnosis [1, 5]. CT is effective in evaluating cortical structures, displacement, and dislocation, and could provide reformatted images from different angles [1, 16]. MRI is also useful in detecting fractures and could identify any associated tissue or ligament injury [1].

The most common causes of isolated medial cuneiform fractures are a direct blow or axial or rotational force applied to the midfoot [1, 4, 5, 7, 12]. Isolated medial cuneiform fractures are usually nondisplaced and stable and are treated conservatively with 6-8 weeks of immobilization in a short leg cast. However, if displaced, reduction and internal fixation are required [12]. However, surgical techniques have not been established. In this case, an isolated medial cuneiform fracture may have been caused by direct impact, such as jumping and landing during a handball game. In addition, misdiagnosis of medial cuneiform fractures and inadequate treatment can lead to non-union.

We performed surgical treatment because the displaced fracture and sclerotic changes were observed, and the bone union was not expected. Open reduction and internal fixation were performed using a compression screw and a compression staple with autologous bone grafts. Recently, there have been several reports of good clinical results using compression staples for bone fixation and osteotomy [17-20]. Russel et al evaluated the biomechanical properties of new shape memory alloy (SMA) staples arranged in different configurations in a repeatable biomechanical integrity and showed SMA staples provide fixation with the ability to dynamically apply and maintain compression across a simulated arthrodesis following a range of loading conditions first tarsometatarsal arthrodesis model [21].

Schipper et al. reported the radiographic union rate of the hindfoot and midfoot arthrodesis using nitinol staples and/or screws. Radiographic union was seen in 93.8% of patients and 95.1% of joints using the nitinol staple construct. Radiographic union was seen in 90.6% of patients and 95.7% of joints using the nitinol combined staple and screw construct [20]. O’Neil et al. showed reported a significant increase in stability of the talonavicular joint when the nitinol staple-plate construct was placed to augment a single cannulated screw for a talonavicular fusion [19].

Screws and staples could provide strong fixation, and staples and screws are light and small, which might be useful in comminuted fractures that are difficult to fix with plates. In this case, the medial cuneiform bone fragment was too small to be fixed with a plate. Fixation using a combination of compression staples and screws could be used in small spaces. Therefore, the use of a compression screw and a compression staple provided strong fixation and sustained pressure on the fracture site, which may have resulted in bone fusion and good clinical results.

## Conclusions

We encountered a case of an isolated medial cuneiform fracture requiring surgical treatment. Medial cuneiform fractures are often treated conservatively, but are often missed because of diagnostic difficulties. Delayed diagnosis may lead to the need for surgical treatment. Since medial cuneiform fractures often have small bone fragments, surgical treatment using a combination of compression staples and screws may be simpler and more useful for obtaining strong fixation.
